# Supplementary material for: Stacking Model‐Based Classifiers for Dealing With Multiple Sets of Noisy Labels
Source: Biom J. 2025 Mar 12;67(2):e70042. doi: 10.1002/bimj.70042 (PMC11898607; doi:10.1002/bimj.70042)
Supplement: Supplementary file 1 — Supporting Information [file BIMJ-67-e70042-s001.zip › density-based-ensemble-model-main/competitors_real_data/netlab-master/netlab-master/help/gmmsamp.htm]

Netlab Reference Manual gmmsamp


# gmmsamp

## Purpose

Sample from a Gaussian mixture distribution.

## Synopsis

```
data = gmmsamp(mix, n)
[data, label] = gmmsamp(mix, n)
```

## Description

`data = gsamp(mix, n)` generates a sample of size `n` from a
Gaussian mixture distribution defined by the `mix` data
structure. The matrix `x` has `n`
rows in which each row represents a `mix.nin`-dimensional sample vector.

`[data, label] = gmmsamp(mix, n)` also returns a column vector of
classes (as an index 1..N) `label`.

## See Also

`gsamp`, `gmm`

---

**Pages:**
Index


---

Copyright (c) Ian T Nabney (1996-9)
